# Effect of dapagliflozin on ferroptosis through the gut microbiota metabolite TMAO during myocardial ischemia–reperfusion injury in diabetes mellitus rats

**DOI:** 10.1038/s41598-024-64909-5

**Published:** 2024-06-15

**Authors:** Lian Wang, Yao Wang, Heng Xu, Wenyuan Li

**Affiliations:** 1 Department of Infectious Diseases, Renmin Hospital of Wuhan University, Wuhan, 430060 Hubei China; 2 College of Medicine, Wuhan University of Science and Technology, Wuhan, 430070 Hubei China; 3 Department of Anesthesiology, Renmin Hospital of Wuhan University, Wuhan, 430060 Hubei China

**Keywords:** Diabetes, Myocardial ischemia/reperfusion, Dapagliflozin, Trimethylamine N-oxide, Ferroptosis, Molecular docking, Computational biology and bioinformatics, Molecular biology, Cardiology, Endocrinology

## Abstract

Dapagliflozin (DAPA) demonstrates promise in the management of diabetic mellitus (DM) and cardiomyopathy. Trimethylamine N-oxide (TMAO) is synthesized by the gut microbiota through the metabolic conversion of choline and phosphatidylcholine. Ferroptosis may offer novel therapeutic avenues for the management of diabetes and myocardial ischemia–reperfusion injury (IRI). However, the precise mechanism underlying ferroptosis in cardiomyocytes and the specific role of TMAO generated by gut microbiota in the therapeutic approach for DM and myocardial IRI utilizing DAPA need to be further explored. Nine male SD rats with specific pathogen-free (SPF) status were randomly divided equally into the normal group, the DM + IRI (DIR) group, and the DAPA group. The diversity of the gut microbiota was analyzed using 16S rRNA gene sequencing. Additionally, the Wekell technique was employed to measure the levels of TMAO in the three groups. Application of network pharmacology to search for intersection targets of DAPA, DIR, and ferroptosis, and RT-PCR experimental verification. Ultimately, the overlapping targets that were acquired were subjected to molecular docking analysis with TMAO. The changes of Bacteroidetes and Firmicutes in the gut microbiota of DIR rats were most significantly affected by DAPA. Escherichia-Shigella and Prevotella_9 within the phylum Bacteroidetes could be identified as the primary effects of DAPA on DIR. Compared with the normal group, the TMAO content in the DIR group was significantly increased, while the TMAO content in the DAPA group was decreased compared to the DIR group. For the network pharmacology analysis, DAPA and DIR generated 43 intersecting target genes, and then further intersected with ferroptosis-related genes, resulting in 11 overlapping target genes. The mRNA expression of ALB, HMOX1, PPARG, CBS, LCN2, and PPARA decreased in the DIR group through reverse transcription polymerase chain reaction (RT-PCR) validation, while the opposite trend was observed in the DAPA group. The docking score between TMAO and DPP4 was − 5.44, and the MM-GBSA result of − 22.02 kcal/mol. It epitomizes the finest docking performance among all the target genes with the lowest score. DAPA could reduce the levels of metabolite TMAO produced by gut microbiota, thereby regulating related target genes to decrease ferroptosis in DIR cardiomyocytes.

## Introduction

Diabetes mellitus (DM) is a complex chronic metabolic disease with multiple factors^1^. Myocardial ischemia/reperfusion injury (IRI) typically manifests in cardiac pathologies, including coronary atherosclerotic heart disease and coronary spasm, and is precipitated by acute myocardial ischemia and hypoxia^2^. Diabetes has a heightened susceptibility to myocardial IRI^3^. The incidence of multivessel coronary disease in diabetic patients with coronary heart disease significantly exceeds that in non-diabetic patients, and myocardial IRI occurs after myocardial ischemia and reperfusion. There is evidence to suggest that individuals with diabetes mellitus exhibit a more pronounced degree of cardiac insufficiency in comparison to those with no diabetes^4,5^. Therefore, the mechanism of reducing the incidence of myocardial IRI in diabetes patients still needs further research.

Numerous investigations conducted recently have revealed that diabetic patients exhibit a clear gut microbiota imbalance^6^. Newly discovered evidence shows the potential clinical significance of gut microbiota in the pathophysiology of cardiomyopathy. Imbalance can lead to cardiac insufficiency and other diseases in patients with cardiomyopathy^7,8^. Growing evidence has demonstrated that interventions directed at gut microbiota may play a critical role in many cardiovascular diseases including heart failure, hypertension, and coronary artery disease^9^. The association between gut microbiota and heart disease with diabetes provides us with new treatment ideas. Trimethylamine N-oxide (TMAO) is a gut microbiota-dependent metabolite produced from choline and phosphatidylcholine^10^. When there is an imbalance in gut microbiota and a decrease in probiotics, the ingestion of choline in the diet can lead to the metabolic conversion of choline by intestinal bacteria, resulting in the production of trimethylamine. Trimethylamine has the potential to undergo hepatic metabolism, leading to the synthesis of TMAO. Previous studies have shown that Firmicutes in the male intestinal flora produce more TMAO than Bacteroidetes^11^. As a metabolite of gut microbiota, TMAO can prevent myocardial infarction and post myocardial infarction posterior ventricular remodeling^12^. The association between TMAO and myocardial IRI is still unclear and needs further exploration.

Dapagliflozin (DAPA) is a Sodium-glucose cotransporter-2 inhibitor, which is an anti-diabetic drug with promising cardiovascular effects^13^. The DAPA alleviates myocardial IRI by reducing ferroptosis^14^. Ferroptosis remains a novel type of programmed cell death characterized by intracellular ferroptosis overload, glutathione depletion^15^, and lipid peroxidation and has been involved in the pathogenesis of various cardiovascular diseases, including myocardial IRI and DM^16,17^.

To further explore the treatment of myocardial IRI’s new target, it is necessary to investigate the intervention mechanism of DAPA on cardiomyocytes ferroptosis through the gut microbiota-TMAO axis in diabetes mellitus myocardial ischemia–reperfusion injury (DIR). This article utilized network pharmacology combined with bioinformatics technology to investigate the mechanism and signaling pathways of DAPA in treating myocardial IRI. Additionally, molecular docking technology and cell assays were employed to validate the obtained results.

## Materials and methods

### Rat model establishment and drug administration

Nine specific pathogen-free (SPF) male Sprague–Dawley (SD) rats (weighing 200–220 g) were purchased from Hunan Slake Jingda Experimental Animal Co., Ltd (Hunan, China), license number SCXK (Hunan) 2019-0004. The experiment was conducted at a temperature of 25 °C ± 2 °C, relative humidity of 50% ± 15%, and normal circadian rhythm (12 h (h) dark/12 h light) in Renmin Hospital of Wuhan University. All the rats got free water and food. The study protocols were by the internationally accepted principles and Guidelines for the Care and Use of Laboratory Animals of Wuhan University [IACUC Issue No. WDRM (welfare) 20230706C], and confirming that all experiments were performed in accordance with relevant guidelines and regulations. All the rats were randomly equally divided into the normal group, the DIR group, and the DAPA group.

The rat models were built as follows. The DM rats were established by injecting 1% streptozotocin into the tail vein at 60 mg per kilogram (mg/kg) dose. After 3 days, if the fasting blood glucose level was higher than 16.7 mmol per liter (mmol/L), the DM model was successfully built. The normal group was given a 0.9% sodium chloride injection. After 8 weeks, the DIR group and DAPA group were treated with myocardial ischemia–reperfusion intervention. Dapagliflozin (40 mg/kg/day) was administrated once a day via intraperitoneal injection over seven days before myocardial IRI surgery for the DAPA group^18^. The rats were given electrocardiogram (ECG) monitoring management. The rats in each group were intubated, connected to a ventilator, and mechanically ventilated at a frequency of 80–90 times/min. The heart rate (HR) and blood pressure were recorded. The rats’ hearts were exposed by sternotomy along the left edge of the sternum. The left anterior descending (LAD) coronary artery between the left atrial appendage and the pulmonary artery cone was sutured and then covered with saline gauze. If the left ventricle apex for myocardial blanched and ST segment of ECG was elevated, the LAD coronary artery was successfully ligatured. After 30 min of ischemia, the ligature was cut to restore the reperfusion of coronary arteries. At the same time, the anterior wall of the left ventricle turned red, and the descending ST segment of the electrocardiogram indicated the successful reperfusion. Then, the coronary arteries restored the reperfusion for 2 h. All the contents of rat intestines were collected and weighed under aseptic conditions, and stored in a refrigerator at − 80 °C for subsequent experiments. The feces were collected for the follow-up experiments. The general conditions for the three group rats are shown in Table 1.

**Table 1:** General condition for the three group rats.

Group	Body weight (g)	Food intake g/(kg days)	Water intake ml/(kg days)	Blood glucose (mmol/L)
Normal group	384.0 ± 26.5	62.5 ± 5.8	112.3 ± 9.2	6.3 ± 0.8
DIR group	211.2 ± 16.3**	105.3 ± 8.6**	206.7 ± 15.8**	25.8 ± 3.2**
DAPA group	289.4 ± 21.8^##^	86.5 ± 7.2^##^	163.1 ± 14.4^##^	16.2 ± 2.8^##^

Data are presented as the means ± S.D. **P < 0.01, compared with normal group rats. ^##^P < 0.01, compared with DIR group rats. *DIR group* diabetes myocardial ischemia/reperfusion injury group, *DAPA group* dapagliflozin treat diabetes myocardial ischemia/reperfusion injury group.

### Reverse transcription polymerase chain reaction (RT-PCR) amplification extraction of total DNA from the microbiome and PCR amplification

The total DNA of the microbiome in feces was extracted, and the DNA extraction quality was detected by agarose gel electrophoresis, and the DNA was quantified by an ultraviolet spectrophotometer^19,20^. The sequence was amplified using the primers in Table 2^21–23^, and the PCR product was confirmed by 2% agarose gel electrophoresis. Ultra-purified water was used for DNA extraction to eliminate the possibility of false positive PCR results as negative controls. The PCR products were purified by AMPure XT beads (Beckman Coulter Genomics, Danvers, MA, USA) and quantified by Qubit (Invitrogen, USA). The amplicon pool was used for sequencing, and the size and quantity of the amplicon library were evaluated on the Agilent 2100 Bioanalyzer (Agilent, USA) and Illumina (Kapa Biosciences, Woburn, MA, USA) library quantification kits. The library was sorted on the NovaSeq PE250 platform. Based on previous literature, the primer sequences for regions Sort the library on the NovaSeq PE250 platform. With reference to previous literature reports, the primer sequences of V3–V4^21^, V4^22^, V4–V5^22^, and Archaeal^23^ were shown are provided in Supplementary Table S1.

**Table 2:** Primer sequences.

Gene	Forward (5*′*–3*′*)	Reverse (5*′*–3*′*)
ALB	5ʹ-GCCGAGAAGCACACAAGAGT-3ʹ	5ʹ-GGGAAAAGGCAATCAGGACT-3ʹ
MAPK1	5′-AACACAACAAAAAGCCGCCC-3′	5′-TGGTACTCAGTGGGGGTGAT-3′
HMOX1	5′-GTCCCAGGATTTGTCCGAGG-3′	5′-GGAGGCCATCACCAGCTTAAA-3′
PPARG	5′-GTCTCACAATGCCATCAGGT	5′-AGCTGGTCGATATCACTGGA-3′
MAPK8	5′-CCGTACATCAACGTCTGGTATGAT-3′	5′-CTCCCTTTCATCTAACTGCTTGTC-3′
PARP1	5′-TCTCCAATCGCT TCTACACCCT-3′	5′-TACTGCTGTCATCAGACCCACC-3′
CBS	5′-CACGAAGTTTAGCAGGTCAATG-3′	5′-GGTCCATGAGCAGATCCAAT-3′
SRC	5′-TGGCGAGAACCTGGTGTGCAA-3′	5′-TTGGCACCTTGCCGAGCTGTT-3′
LCN2	5′-AGCGAATGCGGTCCAGAAAGAAAG-3′	5′-CGAGGATGGAAGTGACGTTGTAGC-3′
PPARA	5′-GATTCGGAAACTGCAGACCTC-3′	5′-TAGGAACTCTCGGGTGATGA-3′
DPP4	5′-ATTCCGTACCCAAAGGCAGG-3′	5′-AGGCCACGTCACACAAGTAG-3′
β-Actin	5′-TGCTATGTTGCCCTAGACTTCG-3′	5′-GTTGGCATAGAGGTCTTTACGG-3′

Primer sequences of various overlapping genes. *ALB* Albumin, *MAPK1* Mitogen-Activated Protein Kinase1, *HMOX1* Heme Oxygenase1, *PPARG* Peroxisome Proliferator-Activated Receptor Gamma, *MAPK8* Mitogen-Activated Protein Kinase8, *PARP1* Poly (ADP-ribose) Polymerase1, *CBS* Cystathionine Beta-Synthase, *SRC* Src kinase, *LCN2* Lipocalin-2, *PPARA* Peroxisome Proliferator-Activated Receptor Alpha, *DPP4* Dipeptidyl Peptidase-4.

### Detection TMAO detection of TMAO

The TMAO level in the heart tissue was detected by the Wekell technique^24,25^. Three groups of heart tissue samples were extracted and subsequently treated with an equimolar combination of disodium Ethylene Diamine Tetraacetic Acid (EDTA) and ferrous sulfate. The sample absorbance for the three groups was measured by biosystems spectrophotometer at 410 nm absorbance. Finally, the TMAO content in each tissue was calculated based on the absorbance of the sample.

### Network pharmacology analysis

The Canonical SMILES of DAPA were accessed on the platform of PubChem (https://pubchem.ncbi.nlm.nih.gov/). And the genes targets for DAPA waswere identified in the databases Swiss Target Prediction (http://www.swisstargetprediction.ch/) and Pharmmappe (http://www.lilab-ecust.cn/pharmmapper/) using the Canonical SMILES. DM and myocardial IRI-related target genes were retrieved from the online human gene database GeneCards (https://www.genecards.org/) Online Mendelian Inheritance in Mans (OMIM) database (https://omim.org/). The targets of DAPA are overlapped with the disease targets genes by using the online tool Venny2.1 (http://bioinfogp.cnb.csic.es/tools/venny/) to predict the potential targets of DAPA in myocardial IRI in patients with diabetes treatment, and then the result was visualized with a Venn diagram. Import all obtained overlapping genes into the STRING (https://cn.string-db.org/) database. Download the protein–protein interaction (PPI) file and import it into Cytoscape 3.10.1 software for visualization processing. Set the size of the nodes in the network graph by the Degree value when creating the PPI network for DAPA, myocardial IRI, and DM. All obtained overlapping genes were to be imported into the STRING (https://cn.string-db.org/) database. The protein–protein interaction (PPI) file was to be downloaded and then imported into Cytoscape 3.10.1 software for visualization processing. When creating the PPI network for DAPA, myocardial IRI, and DM, the size of the nodes in the network graph was to be set by the Degree value. The gene ontology (GO) analysis was predominantly employed to depict the functions of targets, encompassing biological processes (BP), molecular function (MF), and cellular components (CC)^26^. The signaling pathways on the Kyoto Encyclopaedia of Genes and Genomes (KEGG) database were analyzed based on the crucial targets. The drug and disease intersection genes were uploaded to the DAVID database (https://david.ncifcrf.gov/tools.jsp/) for performing GO and KEGG functional enrichment analysis.

### Obtaining overlapping gene genes

Ferroptosis-associated genes were collected from the FerrDb database (http://www.zhounan.org/ferrdb/current/)^27^. Venn diagrams were employed to identify shared targets between gene targets of drug-diseases and ferroptosis-associated genes. These targets serve as common targets between DAPA active ingredient therapy for DIR targets and ferroptosis in cardiomyocytes. The collected targets were visualized using Cytoscape3.10.1 software.

### Detecting mRNA level by reverse transcription polymerase chain reaction (RT-PCR)

Referring to previous methods^28^, the RNAiso Plus kit (Code No. 9108Q, Takara, Dalian, China) was used to extract total RNA from each group of myocardial tissue. PrimeScript™ RT reagent kit (Code NO. 9108Q RR037Q, Takara, Dalian, China) was used to reverse the transcription of total RNA into cDNA used to reverse transcribe the total RNA into cDNA. The process of reverse transcription was carried out at 37 °C for a duration of 15 min, followed by a brief incubation at 85 °C for 5 s. SYBR Premix Ex Taq kit (Cat. No. RR420A, Takara, Dalian, China) was used for qRT PCR. The program settings for PCR were 95 °C for 5 s, followed by 95 °C for 5 s and 60 °C for 34 s for a total of 40 cycles. All genes expression levels were calculated by using the 2^−ΔΔCT^ method. The primer sequence is shown in Table 2.

### Molecular docking

The crystal structure of overlapping target proteins was retrieved from the Research Collaboratory for Structural Bioinformatics (RCSB) Protein Data Bank (PDB) database or AlphaFold2. Apply the Protein Preparation Wizard module of the Schrödinger software to the obtained protein crystals for the following processes: protein preprocess, regenerate states of native ligand, H-bond assignment optimization, protein energy minimization, and removal waters. Process the 2D sdf structure file of the compound TMAO using the LigPrep module in Schrödinger to generate all its 3D chiral conformations. The optimal binding site was predicted by using the SiteMap module in Schrödinger. Then, utilize the Receptor Grid Generation module in Schrödinger to set the most suitable enclosing box that perfectly encapsulates the predicted binding site. Finally, the active sites of the intersection target proteins are obtained. The active sites of the intersection target proteins are molecularly docked with the processed ligand compound TMAO.

The crystal structure of overlapping target proteins was retrieved from the Research Collaboratory for Structural Bioinformatics (RCSB) Protein Data Bank (PDB) database or AlphaFold2. The Protein Preparation Wizard module of the Schrödinger software was applied to the obtained protein crystals for the following processes: protein preprocess, regenerate states of native ligand, H-bond assignment optimization, protein energy minimization, and removal of waters. The 2D sdf structure file of the compound TMAO was processed using the LigPrep module in Schrödinger to generate all its 3D chiral conformations. The optimal binding site was predicted using the SiteMap module in Schrödinger. Subsequently, the Receptor Grid Generation module in Schrödinger was utilized to set the most suitable enclosing box that perfectly encapsulates the predicted binding site. Finally, the active sites of the intersection target proteins were obtained. The active sites of the intersection target proteins were molecularly docked with the processed ligand compound TMAO.

### Data analysis, data processing and statistical analyses

**Figure 1:**
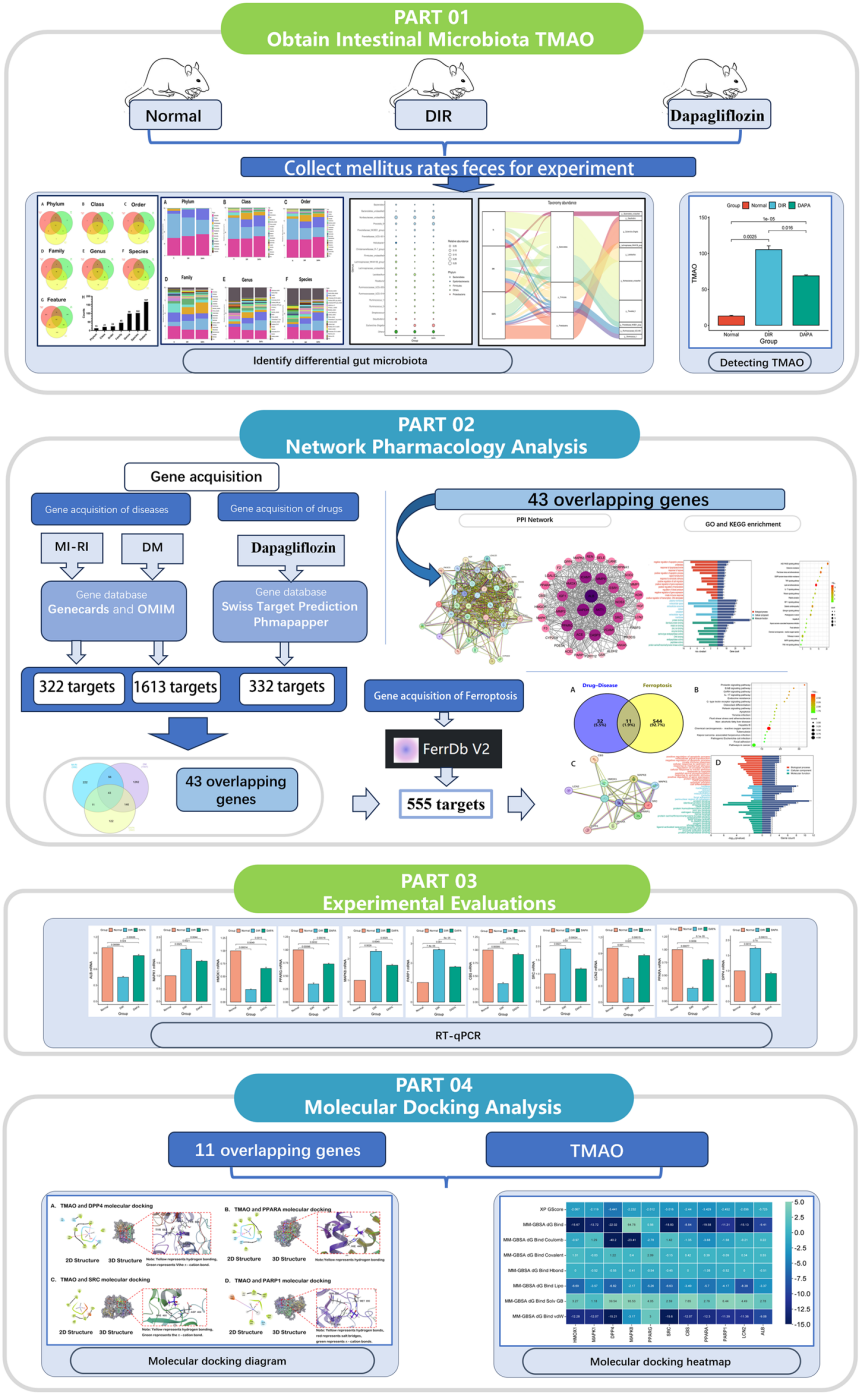
This flowchart divides this study into four parts. The first part identifies the differential microbiota in different groups and measures TMAO values. The second part conducts network pharmacology analysis to identify the intersection targets of DIR, dapagliflozin, and ferroptosis. The third part is RT-PCR validation. The part four docking TMAO with intersecting target molecules.

The manufacturer's recommendations sequenced the samples on the Illumina NovaSeq platform. All samples were sequenced on the Illumina NovaSeq platform. According to the unique barcode of the sample, the paired end sequence was assigned to the sample, and the barcode and primer sequence introduced in the library construction were removed. The matching end to read was merged using FLASH. According to fqtrim (v0.94), the original read data was filtered under specific filtering conditions to obtain high-quality clean tags. The chimeric sequences (v2.3.4) were filtered using Vsearch software. The demodulation and extraction of both the feature table and feature sequence are performed using DADA2. Diversity and diversity are calculated by normalizing to the same random sequence. Then according to the SILVA (release 132) classifier, the relative abundance of each sample was used to normalize the feature abundance. Five indicators were utilized for the analysis of sample species diversity, including Chao1, Observed species, Goods coverage, Shannon, and Simpson, to assess the complexity of alpha diversity. All these indicators in the sample were calculated by using QIIME2. The SPSS 25.0 software was then used to analyze the above statistical data. The results are expressed as mean ± standard deviation. Student's t-test was used to compare the means of two groups or samples. Analysis of Variance (ANOVA) was used when comparing means among three or more groups or samples. P < 0.05 was considered statistically significant. The technical process of the complete article experiment is shown in Fig. 1

### Ethical approval

The animal experiment was approved by the Laboratory Animal Care and Committee of Renmin Hospital of Wuhan University (IACUC Issue No. WDRM (welfare) 20230706C). We confirm that animal research was conducted in accordance with the ARRIVE guidelines.

## Results

### Different expression for microbial species of phylum, class, order, family, genus, species in each group

**Figure 2:**
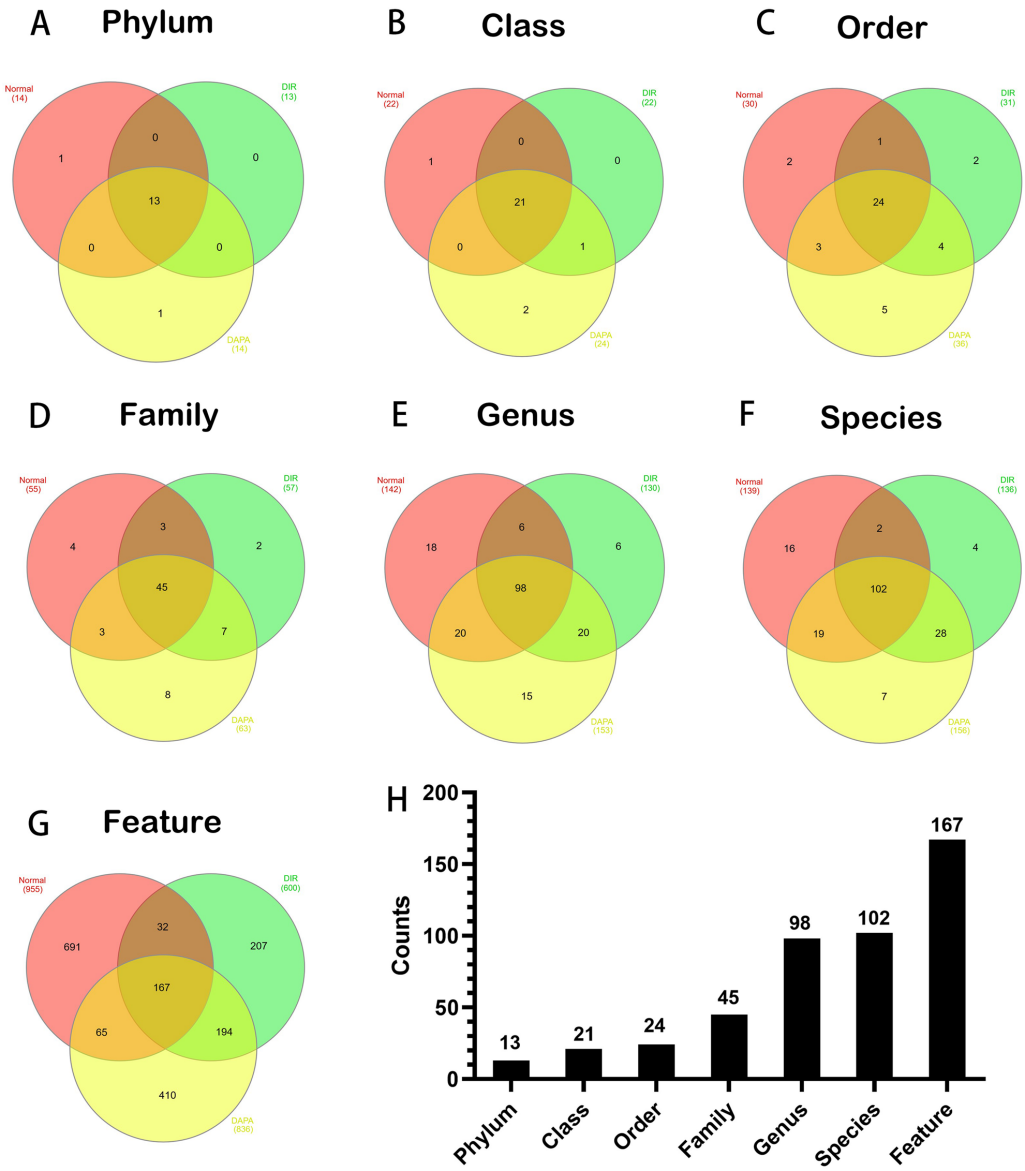
Venn diagram showed the differentially expressed microbial species of Phylum (**A**), Class (**B**), Order (**C**), Family (**D**), Genus (**E**), Species (**F**) in each group. There are 13 species of Phylum microorganisms, 21 species of Class microorganisms, 24 species of Order microorganisms, 45 species of Family microorganisms, 98 species of Genus microorganisms, 102 species of Species microorganisms, 167 species of Feature microorganisms. *N* normal group, *DIR* diabetes myocardial ischemia/reperfusion injury group, *DAPA* dapagliflozin treat diabetes myocardial ischemia/reperfusion injury group.

Table 1 presents the data on the weight, food intake, water intake, and blood glucose levels of the different rat groups. As shown in Fig. 2A–H, the detected bacterial groups in the normal group, the DIR group, and the DAPA group were intersected. The results showed that there were 13 species of Phylum microorganisms, 21 species of Class microorganisms, 24 species of Order microorganisms, 45 species of Family microorganisms, 98 species of Genus microorganisms, 102 species of Species microorganisms, and 167 species of Feature microorganisms.

### Changes of specific gut microbiota in phylum, class, order, family, genus, and species

**Figure 3:**
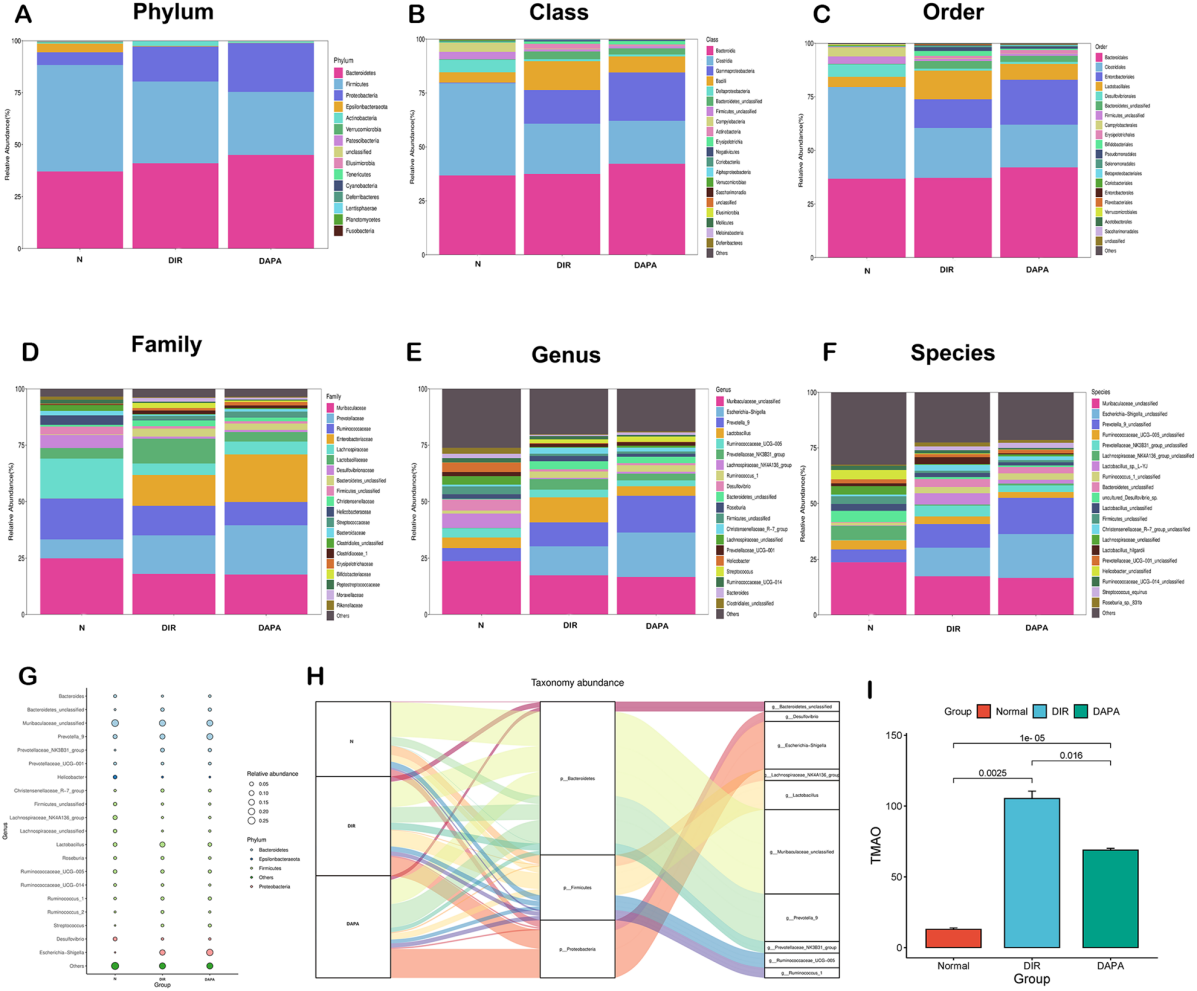
(**A**–**F**) The top 21 changes of specific gut microbiota in Phylum, Class, Order, Family, Genus and Species. (**G**) Bubble plot uses the size and color changes of bubbles to intuitively reflect the data information in the two-dimensional matrix of species annotations and abundance. The bubble plot displays the species annotation information and relative abundance (circle size) at the genus level in different sample groups and displays the species annotation information (circle color) of the species corresponding to the phylum. (**H**) Sankey plots are used to show the "flow" changes of the data, and the width of the branches indicates the size of the flow. The mulberry map shows the relative abundance of the gut microbiota at the phylum level (middle) and genus level (right) corresponding to different samples (left), and visually displays the two levels of species annotation information that are most concerned in the study of gut microbiota diversity. Correspondence, and proportion. (**I**) The TMAO level in the heart tissue was detected by the Wekell technique, increase (P < 0.05) in TMAO levels in the DIR group compared to the normal group. After the dapagliflozin intervention, the TMAO levels in the DAPA group showed a decline (P < 0.05). *N* normal group, *DIR* diabetes myocardial ischemia/reperfusion injury group, *DAPA* dapagliflozin treat diabetes myocardial ischemia/reperfusion injury group.

The Fig. 3A–F shows the top 21 Phylum, Class, Order, Family, Genus, and Species in each group abundance levels in each group of samples. Supplementary Table S2 shows the changes in the top five bacterial groups with the most obvious changes in Phylum, Class, Order, Family, Genus, and Species.

For the Phylum category, the total content of Bacteroidetes, Firmicutes, and Proteobacteria accounted for more than 75%. Compared with the normal group, the abundance value of Bacteroidetes and Proteobacteria in the DIR group was increased (P < 0.05), and the abundance value of Firmicutes was decreased (P < 0.05). Compared with the DIR group, the abundance value of Bacteroidetes and Proteobacteria in the DAPA group was further increased (P < 0.05), and the abundance value of Firmicutes further decreased (P < 0.05).

For the Class category, the total content of Bacteroidia, Clostridia, and Gammaproteobacteria accounted for more than 75%. Compared with the normal group, the abundance value of Bacteroidia and Gammaproteobacteria in the DIR group was increased, the change of Gammaproteobacteria was significant (P < 0.05), and the abundance value of Clostridia decreased (P < 0.05). Compared with the DIR group, the abundance values of Bacteroidia and Gammaproteobacteria in the DAPA group were further increased (P < 0.05), and the abundance values of Gammaproteobacteria further decreased (P < 0.05).

For the Order category, the total content of Clostridiales, Bacteroidales, and Enterobacteriales accounted for more than 75%. Compared with the normal group, the abundance values ​​of Bacteroidales and Enterobacteriales in the DIR group were increased, the changes in Enterobacteriales were significant (P < 0.05), and the abundance values ​​of Clostridiales decreased (P < 0.05). Compared with the DIR group, the abundance value of Bacteroidales and Enterobacteriales in the DAPA group was further increased (P < 0.05), and the abundance value of Enterobacteriales further decreased (P < 0.05).

For the Family category, the total content of Muribaculaceae, Ruminococcaceae, and Prevotellaceae accounted for more than 45%. Compared with the normal group, the abundance value of Prevotellaceae in the DIR group was increased (P < 0.05), and the abundance value of Muribaculaceae and Ruminococcaceae decreased (P < 0.05). Compared with the DIR group, the abundance value of Prevotellaceae in the DAPA group was further increased (P < 0.05), and the abundance value of Muribaculaceae and Ruminococcaceae further decreased, and the change in Ruminococcaceae was significant (P < 0.05).

For the Genus category, the total content of Muribaculaceae, Escherichia-Shigella, and Prevotella_9 accounted for more than 40%. Compared with the normal group, the abundance value of Escherichia-Shigella and Prevotella_9 in the DIR group was increased (P < 0.05), and the abundance value of Muribaculaceae decreased (P < 0.05). Compared with the DIR group, the abundance value of Escherichia-Shigella and Prevotella_9 in the DAPA group was further increased (P < 0.05), and the abundance value of Muribaculaceae further decreased, and the change was not significant.

For the Species category, the total content of Muribaculaceae, Prevotella_9, and Escherichia-Shigella accounted for more than 40%. Compared with the normal group, the abundance value of Escherichia-Shigella and Prevotella_9 in the DIR group was increased (P < 0.05), and the abundance value of Muribaculaceae decreased (P < 0.05). Compared with the DIR group, the abundance value of Escherichia-Shigella and Prevotella_9 in the DAPA group was further increased (P < 0.05), and the abundance value of Muribaculaceae further decreased, but there was no statistical significance.

### Advanced analysis of gut microbiota

The utilized changes for the bubble plot in bubble size and color could be visually represented by the different data information in the two-dimensional matrix of species annotations and abundance. The bubble plot visually displayed the species annotation information and relative abundance, represented by the size of the circles, at the genus level across various sample groups. Additionally, it indicated the species annotation information, represented by the color of the circles, corresponding to the phylum. As depicted in Fig. 3G, Bacteroidetes and Firmicutes were the two Phyla that exhibited the most prominent changes. Among them, Muribaculaceae and Prevotella_9 displayed the highest content within Bacteroidetes, with variations observed among each component. The content of Lactobacillus within Firmicutes was the highest, with no significant changes observed between groups.

Sankey plots were used to showcase the "flow" changes of the data, and the width of the branches indicated the size of the flow. The mulberry map displayed the relative abundance of gut microbiota at the phylum level (middle) and genus level (right) corresponding to different samples (left). It highlighted the connection and proportions of the two layers of species annotation data that were crucial for understanding the diversity of the gut microbiota. Figure 3H revealed significant changes in Bacteroidetes, Firmicutes, and Proteobacteria at the phylum level. Among them, the corresponding genera of Bacteroidetes were Bacteroidetes, Muribaculaceae, Prevotella_9, and Prevotellaceae_NK3B31. The corresponding genera of Firmicutes included Lachnospiraceae_NK4A136, Lactobacillus, Ruminococcaceae_UCG-005, and Ruminococcusib_1, while Proteobacteria displayed correspondence with Shigella.

### Decreased TMAO levels after DAPA intervening for DIR rats

The differential gut microbiota such as Bacteroidetes and Firmicutes were discovered through advanced analysis, which could produce metabolites TMAO^29^. The changes in TMAO levels after DIR injury and DAPA intervention were further investigated. By measuring TMAO levels, the Fig. 3I showed a significant increase (P < 0.05) in TMAO levels in the DIR group compared to the normal group. However, the TMAO levels in the DAPA group were decreased (P < 0.05). Based on the above results, DAPA was verified to have a protective effect on the myocardium by reducing TMAO levels after DIR.

### Network pharmacology analysis

**Figure 4:**
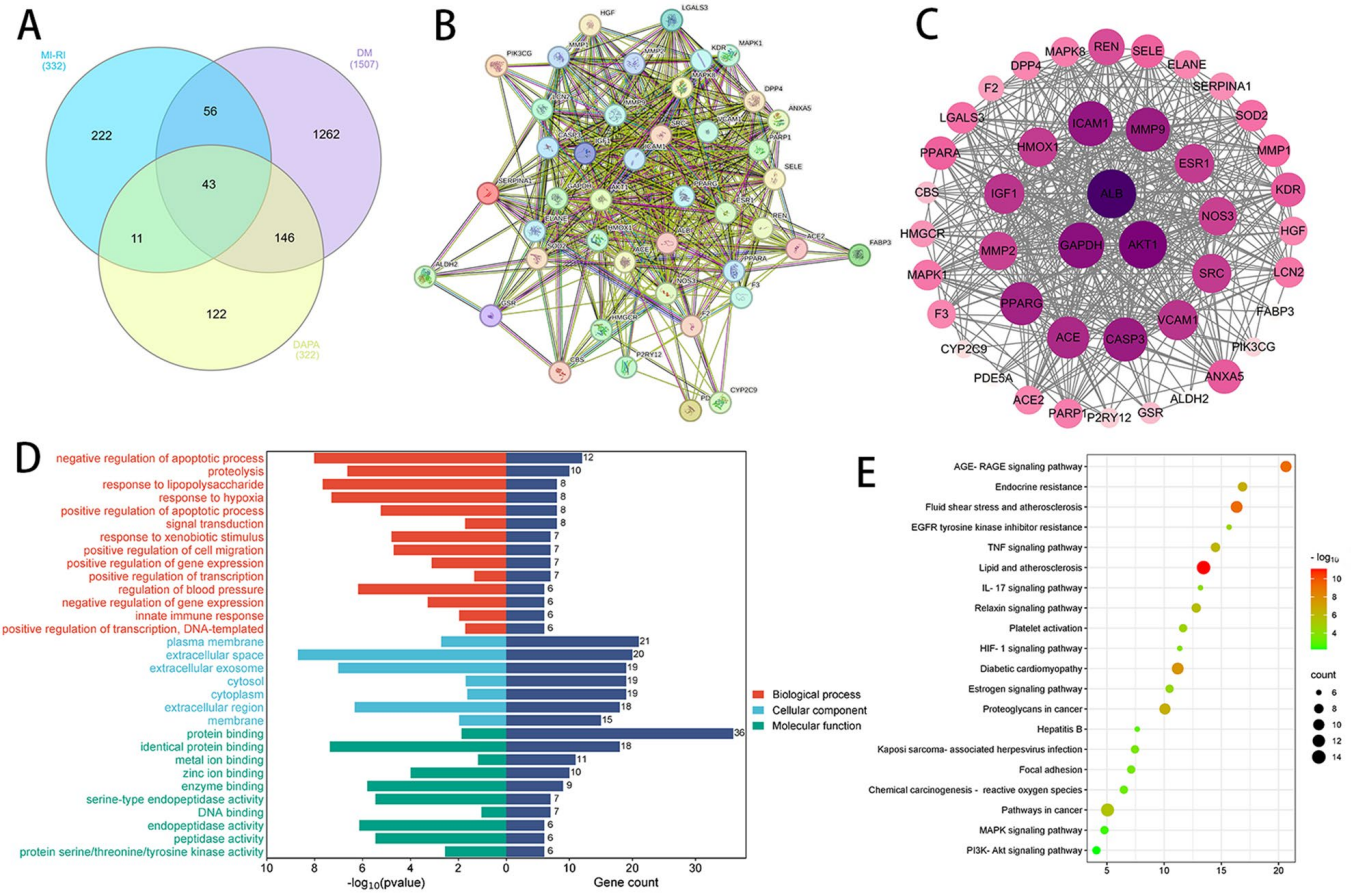
(**A**) The number of 43 overlapping targets between Myocardial IRI (332), DM (1507), and DAPA(322). (**B**) Import all obtained overlapping 43 genes into the STRING database. (**C**) Import it into Cytoscape 3.10.1 software for visualization processing. Build the PPI network of dapagliflozin, myocardial ischemia–reperfusion injury and diabetes mellitus, and set the size of nodes in the network graph according to the Degree value. (**D**) The abscissa left side of the GO bar chart represents the size of the P-value, and the abscissa right side represents gene count. The ordinate represents the names of enriched biological processes, cell components, and molecular functions. (**E**) The bubble size represents the proportion of genes on each pathway. The bubble color represents the degree of enrichment.

The network pharmacology analysis was conducted to further investigate the impact of DAPA on DIR at the genetic level. To get a total of 322 gene targets, locate the gene targets for DAPA in the Swiss Target Prediction and Pharmmapper databases, combine the results, and eliminate duplicates. A total of 322 gene targets were located for DAPA in the Swiss Target Prediction and Pharmmapper databases, combined, and duplicates were eliminated. DM and myocardial IRI-related genes were obtained from the OMIM and GeneCards databases, merged, and removed duplicates to ultimately obtain 1513 DM-related gene targets and 322 myocardial IRI related gene targets. By creating a Venn diagram that combines drug–disease targets, a total of 43 genes were identified at the intersection of drug–disease interactions (Fig. 4A). The intersection genes of drug–disease were uploaded to the STRING database, and potential target genes were predicted using the PPI network, focusing on the human species. The PPI network was then imported into Cytoscape to calculate the degree of network nodes. Genes with scores greater than the average score were selected as key target genes based on their degree ranking. Ultimately, a total of 3 key target genes were identified in Fig. 4B,C.

The 43 candidate targets were analyzed for Gene Ontology biological function and KEGG pathway enrichment. Gene Ontology enrichment analysis was performed based on the cellular components, biological processes, and molecular function. P < 0.05 were analyzed in three groups. Targets were mainly enriched in various biological processes, including negative regulation of the apoptotic process, proteolysis, response to lipopolysaccharide, response to hypoxia, positive regulation of the apoptotic process, and signal transduction. The results showed that targets were significantly enriched in the plasma membrane, extracellular space, extracellular exosome, cytosol, cytoplasm, extracellular region, and membrane in the cellular component subgroup. In terms of molecular function, targets were involved in protein binding, identical protein binding, metal ion binding, zinc ion binding, enzyme binding, serine-type endopeptidase activity, and DNA binding. Based on the KEGG functional enrichment analysis, the results revealed enrichment mainly in the AGE-RAGE signaling pathway in diabetic complications, endocrine resistance, fluid shear stress and atherosclerosis, EGFR tyrosine kinase inhibitor resistance, and TNF signaling pathway in Fig. 4D,E.

### Intersection of ferroptosis targets and DAPA treatment for DIR

**Figure 5:**
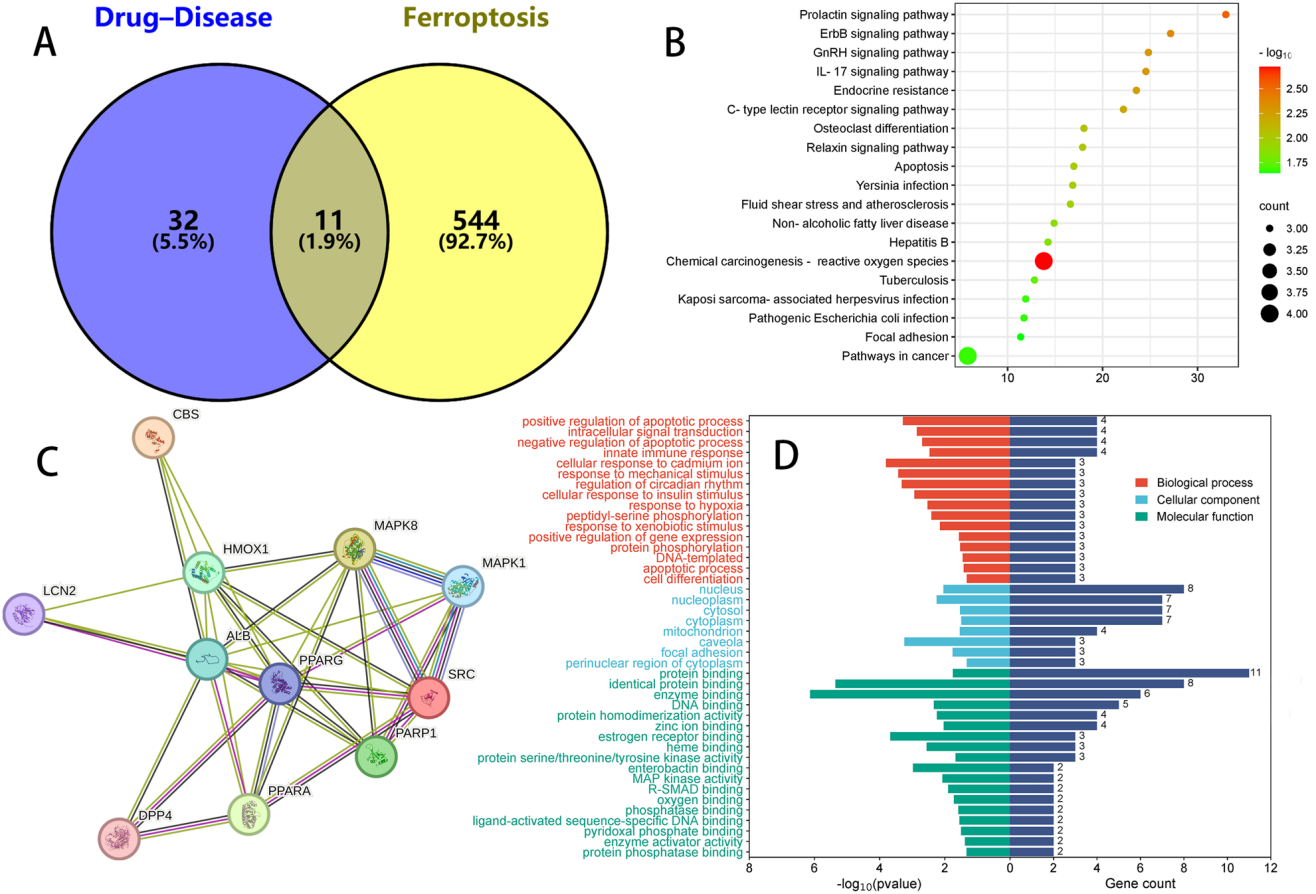
(**A**) The number of 11 overlapping targets between Drug-Disease (43) and Ferroptosis (322). (**B**) The bubble size represents the proportion of genes on each pathway. The bubble color represents the degree of enrichment. (**C**) Import all obtained overlapping 11 genes into the STRING database. (**D**) The abscissa left side of the GO bar chart represents the size of the P-value, and the abscissa right side represents gene count. The ordinate represents the names of enriched biological processes, cell components, and molecular functions.

Importing the 43 common targets of DAPA and DIR, and the 555 ferroptosis targets, obtained 11 overlapping targets (Fig. 5A). The intersection genes of drug–disease were uploaded to the STRING database, and potential target genes were predicted using the PPI network (Fig. 5C). The STRING bioinformatics database was utilized for GO enrichment analysis (Fig. 5D) and signal pathway enrichment (Fig. 5B) at intersection targets. Importing this information these results into Cytoscape and sorting them based on Degree values (Supplementary Table S3). Albumin (ALB), Heme Oxygenase-1 (HMOX1), and Peroxisome Proliferator-Activated Receptor Gamma (PPARG) were found to have higher degree values as key target genes.

### DAPA intervention affects mRNA expression

**Figure 6:**
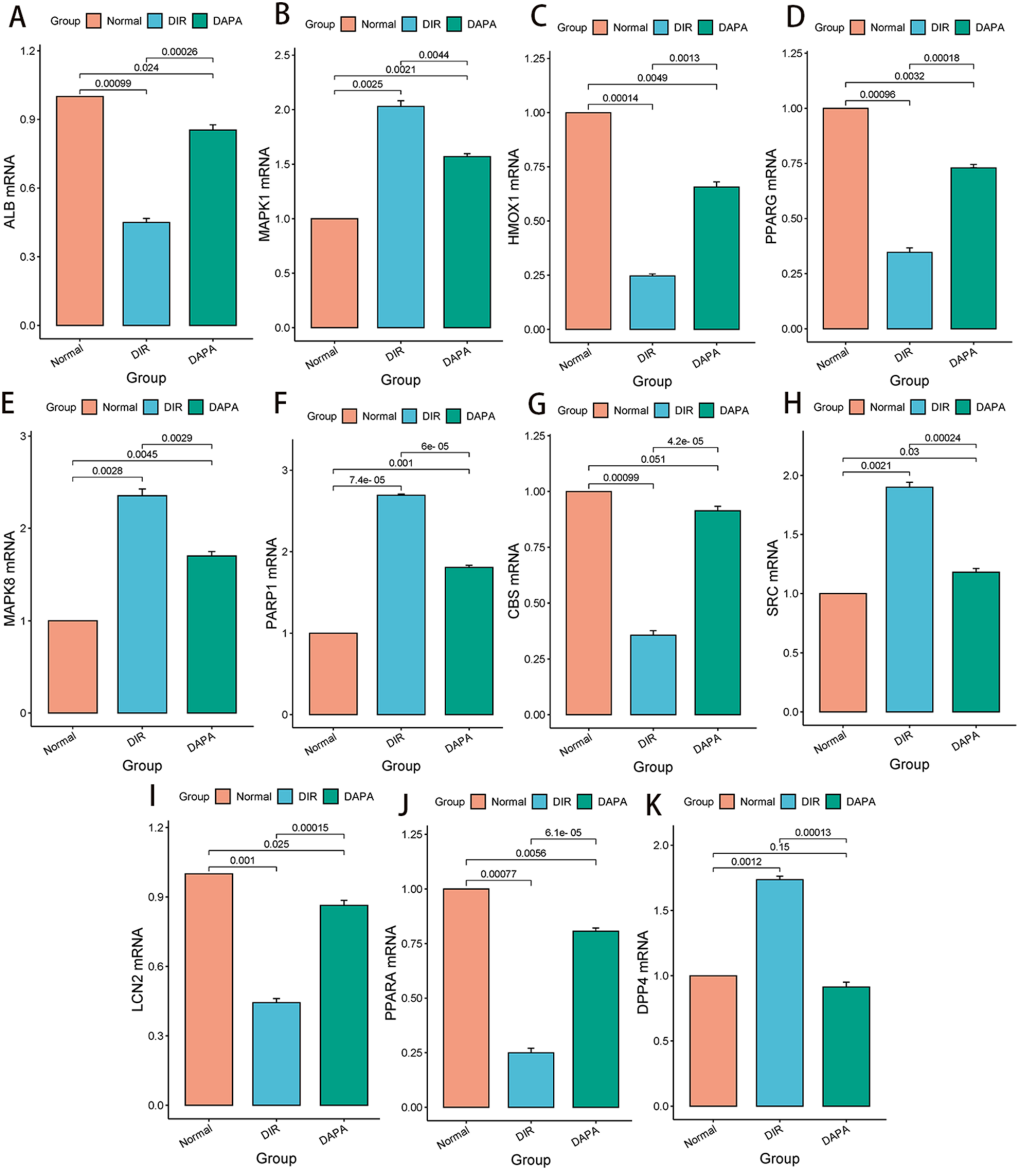
The influence of ALB (**A**), MAPK1 (**B**), HMOX1 (**C**), PPARG (**D**), MAPK8 (**E**), PARP1 (**F**), CBS (**G**), SRC (**H**), LCN2 (**I**), PPARA (**J**), DPP4 (**K**) genes on mRNA expression. *N* normal group, *DIR* diabetes myocardial ischemia/reperfusion injury group, *DAPA* dapagliflozin treat diabetes myocardial ischemia/reperfusion injury group.

Compared with the normal group, the mRNA expression of Albumin (ALB), Heme Oxygenase-1 (HMOX1), Peroxisome Proliferator-Activated Receptor Gamma (PPARG), Cystathionine Beta-Synthase (CBS), Lipocalin-2 (LCN2) and Peroxisome Proliferator-Activated Receptor Alpha (PPARA) in the DIR group was decreased (P < 0.05) through RT-PCR validation. The mRNA expression of Mitogen-Activated Protein Kinase1 (MAPK1), Mitogen-Activated Protein Kinase8 (MAPK8), Poly (ADP-ribose) Polymerase1 (PARP1), Src Tyrosine Kinase (SRC) and Dipeptidyl Peptidase4 (DPP4) in the DIR group was increased (P < 0.05) through RT-PCR validation. While the opposite trend was observed after DAPA intervention in the DAPA group, as shown in Fig. 6.

### Docking analysis of TMAO and intersecting target molecules

**Figure 7:**
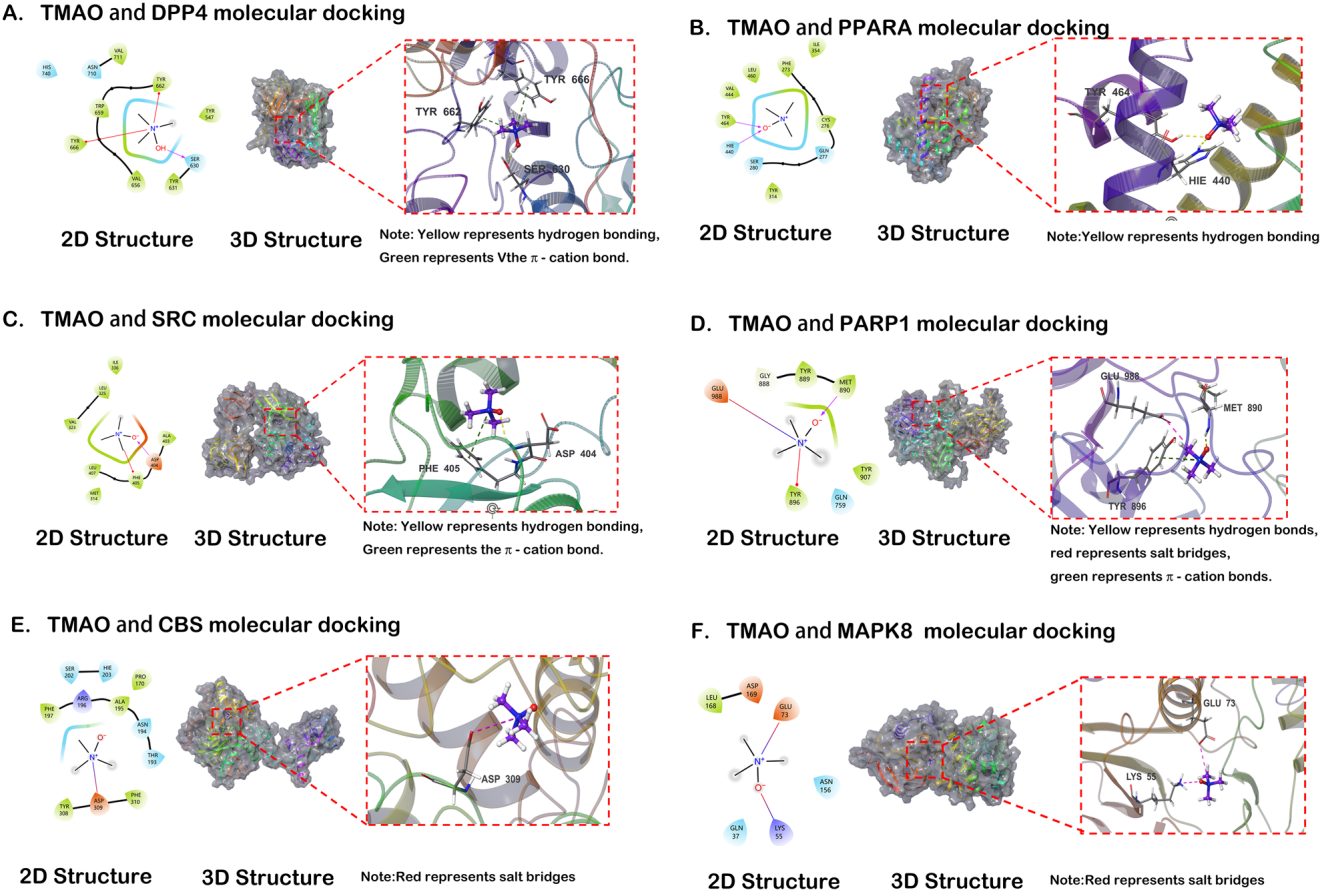
The two-dimensional and three-dimensional structural diagrams of the docking interaction between TMAO and DPP4 (**A**), PPARA (**B**), SRC (**C**), PARP1 (**D**), CBS (**E**), MAPK8 (**F**).

**Figure 8:**
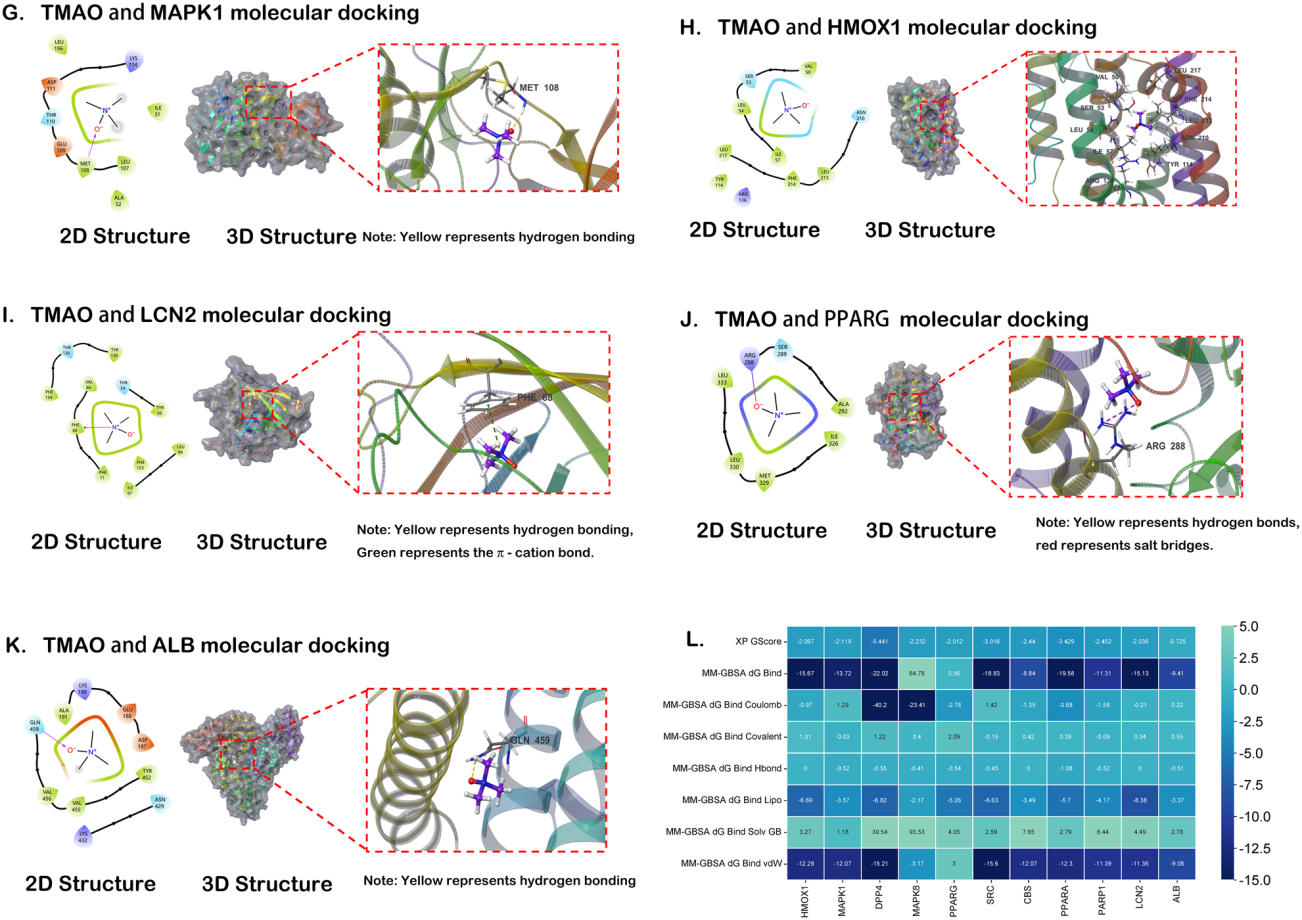
The two-dimensional and three-dimensional structural diagrams of the docking interaction between TMAO and MAPK1 (**G**), HMOX1 (**H**), LCN2 (**I**), PPARG (**J**), ALB (**K**). (**L**) The abscissa represents the core gene, and the ordinate represents the connection method with TMAO. The numbers in the box represent the docking affinity score. The grading color scale ranges from light blue to dark blue. The closer the color is to dark blue, the lower the score and the stronger the binding.

The refined ligand molecule TMAO was docked with the active binding sites of each of the 11 proteins (adopting the highest precision XP docking). A lower score indicates a lower binding free energy and higher binding stability between the compound and the protein. A lower score means that the chemical and protein have a stronger binding stability and a lower binding free energy. The MM-GBSA calculation analysis was performed on the active sites of ligand compound TMAO and 11 proteins. MM-GBSA dG Bind could approximately represent the binding free energy of small molecules with proteins. The lower the binding free energy, the higher the binding stability of ligand TMAO with proteins. Table 3 shows that by integrating XP docking and MM-GBSA results, the docking performance of TMAO with DPP4 was the best, with a docking score of − 5.44 and an MM-GBSA result of − 22.02 kcal/mol. The lower binding free energy suggests a stable binding between TMAO and DPP4. In contrast, the docking scores and binding free energies of TMAO with PPARA and SRC were relatively high, indicating a less stable binding between TMAO and these proteins, as shown in Figs. 7 and 8.

**Table 3:** Molecular docking results.

Title	Compound	Target	XP Gscore	MM-GBSA dG Bind (kcal/mol)
DPP4_6B1E—prepared 1145	TMAO	DPP4	− 5.44	− 22.02
PPARA_7E5I—prepared 1145		PPARA	− 3.43	− 19.58
SRC_4K11—prepared 1145		SRC	− 3.02	− 18.83
PARP1		PARP1	− 2.45	− 11.31
CBS		CBS	− 2.44	− 8.84
MAPK8		MAPK8	− 2.23	64.78
MAPK1 _8AOJ—prepared 1145		MAPK1	− 2.12	− 13.72
HMOX1_6EHA—prepared 1145		HMOX1	− 2.07	− 15.67
LCN2		LCN2	− 2.04	− 15.13
PPARG		PPARG	− 2.01	0.56
ALB		ALB	− 0.73	− 9.41

This table displays the molecular docking results. The MM-GBSA calculation analysis was performed on the active sites of ligand compound TMAO and 11 proteins. MM-GBSA dG Bind could approximately represent the binding free energy of small molecules with proteins. The lower the binding free energy, the higher the binding stability of ligand TMAO with proteins.

## Discussion

The parasitic microorganisms maintain an ecological balance with the host body. They play an important role in maintaining the integrity of the gut microbiota in the host's intestinal epithelial layer, the development and maturation of intestinal mucosal immunity, the regulation of adaptive immunity, and the maturity of the enteric nervous system^30^. The gut microbiota of diabetic patients has undergone significant changes compared with non-diabetic patients^31^. The augmentation of Bifidobacterium bacteria within the gastrointestinal tract of rats subjected to prebiotic supplementation is associated with improved glucose tolerance in said rats. Additionally, it has been found to have a beneficial effect on the reduction of inflammation^32^. Lactobacillus microorganisms are not only related to obesity, they have also been found to regulate the immune response in the intestinal tract^33,34^. The imbalance of gut microbiota may be one of the reasons for the occurrence of coronary heart disease^35^. Individuals diagnosed with inflammatory bowel disease exhibit an elevated susceptibility to coronary heart disease, potentially attributable to compromised intestinal barrier function, heightened intestinal mucosal permeability, and the migration of intestinal bacteria into the bloodstream, thereby contributing to the development of atherosclerosis^36^. TMAO is a gut microbiota-dependent metabolite produced from choline and phosphatidylcholine^10^. Elevated concentrations of TMAO in both the plasma and cerebrospinal fluid have been demonstrated to play a significant role in the emergence of risk factors and actively facilitate the pathogenesis of metabolic, cardiovascular, and cerebrovascular disorders^37–39^. When there is an imbalance in gut microbiota and a decrease in probiotics, the ingestion of choline in the diet can lead to the metabolic conversion of choline by intestinal bacteria, resulting in the production of trimethylamine. Trimethylamine has the potential to undergo hepatic metabolism, leading to the synthesis of TMAO. The latter condition is not only strongly linked to the progression of atherosclerosis but also substantially elevates the likelihood of experiencing major unfavorable cardiovascular events^40^. Numerous investigations have substantiated the association between ferroptosis and the pathogenesis and progression of various illnesses, including cancer and diabetes^41^. Reducing the occurrence of ferroptosis reduces the risk of DIR. Inhibition of DNA methyltransferase 1 could reduce ferroptosis during DIR. This inhibition may influence the process of nuclear receptor coactivator 4-mediated ferritinophagy^42^. Inhibition of ferroptosis in DIR could reduce endoplasmic reticulum stress and myocardial injury^43^. Taken together, the TMAO of gut microbiota and ferroptosis have important effects in on DIR. However, there is currently no research indicating that TMAO of gut microbiota can affect DIR through ferroptosis. In order to seek new therapeutic targets for DIR, it is necessary to effectively intervene in the gut microbiota and its metabolites and identify key targets that affect ferroptosis.

The efficacy of the sodium-glucose co-transporter-2 (SGLT-2) inhibitor DAPA in enhancing cardiovascular outcomes among individuals diagnosed with type 2 diabetes has been substantiated through clinical research^44^. The DAPA provides protection to the myocardial IRI in diabetic rat hearts through the regulation of endothelial nitric oxide synthase and inducible nitric oxide synthase expression, as well as the inhibition of cardiac lipid peroxidation^45^. The DAPA has an impact on the gut microbiota of patients with cardiovascular and kidney diseases^46,47^. However, there is no research indicating the effect of DAPA on the metabolic product TMAO produced by the intestinal microbiota, especially during the period of DIR. SGLT-2 played a renoprotective role in diabetic Kidney disease, at least in part, through alleviating Hypoxia-inducible factor 1 alpha/Heme oxygenase 1-mediated ferroptosis^48^. SGLT-2 restored Glutathione Peroxidase 4 expression in high glucose-treated C2C12 cells, thereby suppressing ferroptosis and promoting cell viability^49^. However, there are few studies on ferroptosis and mechanism exploration of DAPA in DIR. Therefore, it is necessary to carry out research on the effect of TMAO produced by DAPA through gut microbiota on cardiomyocytes ferroptosis in DIR to fill the blank of the research.

From the number of microbial groups contained in each gut sample at the phylum classification level, it could be found that Bacteroidetes and Firmicutes are the most abundant ones, accounting for more than 80% of the total microbes. Compared with the rats in the normal group, the two model groups showed a decrease in the proportion of Firmicutes and an increase in the proportion of Bacteroidetes. Compared with mellitus rats in the DIR group, the proportion of Firmicutes and Bacteroides was further decreased in the DAPA group. This shows that DAPA makes significant changes in the number of gut microbiota, especially Bacteroidetes and Firmicutes.

The diabetes model had the opposite effect on the proportion of Bacteroidetes and Firmicutes. It could be inferred that the body maintains high blood sugar levels in the diabetic state, and the metabolic state such as the inhibition of the conversion of sugar to fat was also reflected in the intestinal microbial abundance level. Further refinement to the level of genus classification, compared with the normal group, the intestinal microbes of the DIR group showed a significant reduction of Muribaculaceae (belonging to the Bacteroidetes phylum), Escherichia-Shigella and Prevotella_9 (both belonging to the Bacteroidetes phylum). In the intestinal microbes of rats in the DAPA group, there was a further decrease of Muribaculaceae microorganisms (but the change was not statistically significant), and Escherichia-Shigella and Prevotella_9 further increased. In each sample, the abundance of microorganisms belonging to the genera Muribaculaceae, Escherichia-Shigella, and Prevotella_9 were among the best among the microorganisms of the phylum Bacteroidetes. The microorganisms belonging to the genus Muribaculaceae are inhibited, and microorganisms belonging to the genus Escherichia-Shigella and Prevotella_9 are further increased in the DAPA group. It plays a decisive role in group changes. In summary, affected by DAPA, the changes of Bacteroidetes and Firmicutes in the gut microbiota of diabetic rats are the most significant. Among them, Escherichia-Shigella and Prevotella_9 in Bacteroidetes can be used as the major effects of DAPA.

The multiple microbiota in the gut microbiota are involved in the metabolic processes of TMAO^50–52^, including the differential microbiota found in this study: the microbiota of Bacteroidetes and Firmicutes. After experimental verification, it was found that the TMAO of the DIR group increased compared to the normal group, while the TMAO of the DAPA group decreased. Therefore, this discovery further proves that DAPA has a significant protective effect in DIR through the TMAO axis of the gut microbiota.

To further investigate the mechanism of ferroptosis in DIR, a network pharmacology analysis was conducted. By creating a Venn diagram that combines DAPA–DIR targets, a total of 43 genes were identified at the intersection of DAPA–DIR interactions. The analysis of GO biological process enrichment reveals that the BP primarily emphasizes the negative regulation of the apoptotic process, proteolysis, and response to lipopolysaccharide. The CC primarily emphasizes the plasma membrane, extracellular space, and extracellular exosomes. The MF predominantly centers around protein binding, identical protein binding, and metal ion binding. Furthermore, The 11 overlapping targets were obtained after crossing with the ferroptosis database, include ALB, MAPK1, HMOX1, PPARG, MAPK8, PARP1, CBS, SRC, LCN2, PPARA, DPP4. The core targets with the highest score were identified by utilizing the PPI network, which include ALB and PPARG. Albumin serves as a carrier protein for sphingosine-1-phosphate (S1P), exhibiting a protective effect on the cardiovascular system by inhibiting oxidative stress, stabilizing cell membranes, and protecting cellular structures^53^. Furthermore, The PPARG agonists possess the ability to safeguard the myocardium against ischemia–reperfusion injury through mechanisms including the inhibition of inflammatory responses, mitigation of oxidative stress, and promotion of angiogenesis^54^. DPP4 possesses the capacity to facilitate the infiltration of inflammatory cells and the secretion of inflammatory mediators, including tumor necrosis factor-alpha (TNF-α) and interleukin-6 (IL-6), thus culminating in inflammatory detriment to cardiomyocytes. Alogliptin, the DPP4 inhibitor, suppresses IRI via adenosine receptors and CREB-dependent signaling pathways^55^. In addition, HMOX1, CBS, LCN2, and PPARA have inhibitory effects on the occurrence and progression of diseases, while MAPK1, MAPK8, PARP1, SRC, and DPP4 have promoting effects on the development of diseases.

Molecular docking technology is a commonly used tool in computer aided drug design. It is frequently used to investigate the areas of interaction between small molecules and macromolecules, which helps to identify the lead compound with the greatest activity and to gather proof for the optimization of its structural design^56^. Intersecting targets, which were the intersection between the targets of cardiomyocytes ferroptosis and the targets of DAPA treatment for DIR, were subjected to molecular docking with TMAO produced by different gut microbiota. The docking performance of TMAO with DPP4 was the best, with a docking score of − 5.441 and an MM-GBSA result of − 22.02 kcal/mol by integrating XP docking and MM-GBSA analysis. The lower binding free energy suggests a more stable interaction between TMAO and DPP4. TMAO exhibits the ability to deeply infiltrate the active pocket of the DPP4 protein, establishing hydrophobic contacts with residues including TYR631, VAL656, TYR666, TRP659, and TYR662. TMAO forms a hydrogen bond with the SER630 residue. In addition, it engages in the formation of π-cation bonds with residues TYR666 and TYR662.

This groundbreaking study explores the phenomenon of ferroptosis caused by TMAO, a metabolite produced by the gut microbiota, in cardiomyocytes of rats with DIR. It was found that DAPA could reduce the content of TMAO and thereby alleviate ferroptosis in cardiomyocyte of rats with DIR. Molecular docking validation was conducted for the effects of TMAO and DAPA on ferroptosis in cardiomyocytes of rats with DIR. The findings of this study provide a solid theoretical foundation for drug indications and future clinical applications. In this study, although the molecular docking between the genes of ferroptosis in cardiomyocytes of DIR rats and the TMAO produced by intestinal microbiota was carried out, in-depth mechanism research has not been effectively validated. Subsequently, the present study will be improved and perfected through the integration of chemical expertise.

## Conclusion

**Figure 9:**
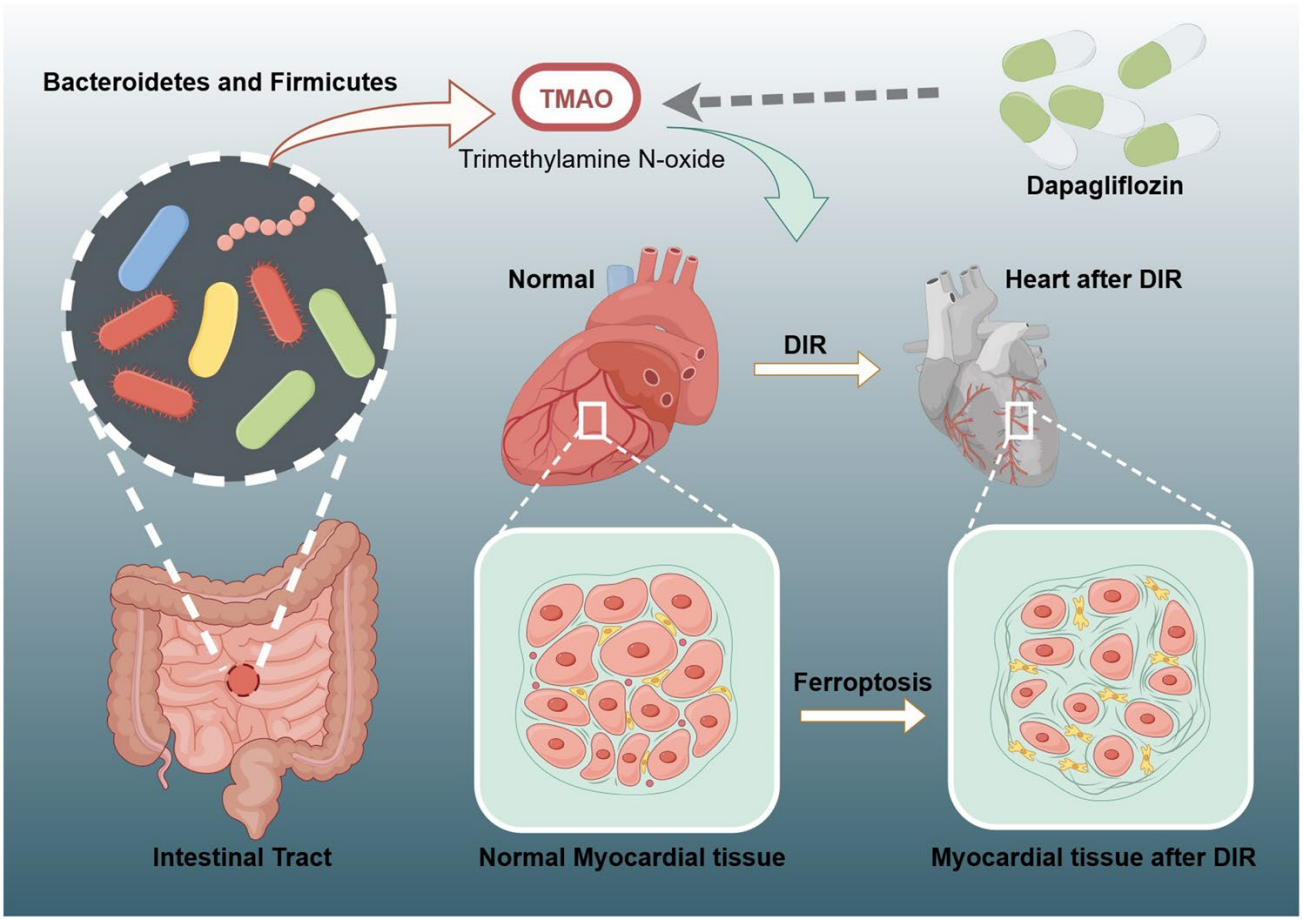
Dapagliflozin could reduce the levels of metabolite TMAO produced by gut microbiota, thereby regulating related target genes to decrease ferroptosis in DIR cardiomyocytes.

Affected by DAPA, the changes of Bacteroidetes and Firmicutes in the gut microbiota of DIR rats are the most significant. Among them, Escherichia-Shigella and Prevotella_9 in Bacteroidetes can be used as the major effects of DAPA on DIR. Compared with the normal group, the TMAO content in the DIR group showed a significant increase, while the TMAO content in the DAPA group decreased compared to the DIR group. After network pharmacology analysis, DAPA and DIR generated 43 intersecting target genes, and then intersected with ferroptosis, resulting in 11 target genes. Through RT-PCR validation, it was found that the expression of ALB, HMOX1, PPARG, CBS, LCN2, and PPARA decreased in the DIR group, while the opposite trend was observed after DAPA intervention. By integrating XP docking and MM-GBSA results, the docking performance of TMAO with DPP4 is the best, with a docking score of − 5.44 and an MM-GBSA result of − 22.02 kcal/mol. In summary, the gut microbiota and its metabolite TMAO could potentially serve as a potential therapeutic target for the treatment of DIR with the use of DAPA. Specifically, TMAO influences the function of ALB, PPARG, and HMOX1 genes in the process of myocardial ferroptosis. DAPA could reduce the levels of metabolite TMAO produced by gut microbiota, thereby regulating related target genes to decrease ferroptosis in DIR cardiomyocytes. The research process is shown in Fig. 9. Therefore, this research presents innovative scientific findings that provide support for the prospective therapeutic application of DAPA in the management of DIR.

### Supplementary Information

Supplementary Table S1.

Supplementary Table S2.

Supplementary Table S3.

## Data Availability

All datasets generated for this study are included in the article.

## Abbreviations

IRI: Ischemia/reperfusion injury
DM: Diabetes mellitus
DIR: Diabetes ischemia/reperfusion injury
DAPA: Dapagliflozin
TMAO: Trimethylamine N-xide
ECG: Electrocardiogram
HR: Heart rate
LAD: Left anterior descending
GO: Gene ontology
MF: Molecular function
BP: Biological processes
CC: Cellular components
KEGG: Kyoto encyclopaedia of genes and genomes
ALB: Albumin
MAPK1: Mitogen-activated protein kinase1
HMOX1: Heme oxygenase1
PPARG: Peroxisome proliferator-activated receptor gamma
MAPK8: Mitogen-activated protein kinase8
PARP1: Poly (ADP-ribose) polymerase1
CBS: Cystathionine beta-synthase
SRC: Src kinase
LCN2: Lipocalin-2
PPARA: Peroxisome proliferator-activated receptor alpha
DPP4: Dipeptidyl peptidase-4
