# Supplementary material for: Effect of dapagliflozin on ferroptosis through the gut microbiota metabolite TMAO during myocardial ischemia–reperfusion injury in diabetes mellitus rats
Source: Sci Rep. 2024 Jun 15;14:13851. doi: 10.1038/s41598-024-64909-5 (PMC11180094; doi:10.1038/s41598-024-64909-5)
Supplement: Supplementary file 1 — Supplementary Table S1. [file 41598_2024_64909_MOESM1_ESM.docx]

| **Primer sequences** | |
| --- | --- |
| **Amplified fragment** | **Primer sequences** |
| V3-V4 | 341F (5'-CCTACGGGNGGCWGCAG-3')  805R (5'-GACTACHVGGGTATCTAATCC-3') |
| V4 | 515F (5'-GTGYCAGCMGCCGCGGTAA-3')  806R (5'- GGACTACHVGGGTWTCTAAT-3') |
| V4-V5 | F (5’-GTGCCAGCMGCCGCGG-3’)  R (5’-CCGTCAATTCMTTTRAGTTT-3’) |
| Archaeal | F (5’-GYGCASCAGKCGMGAAW-3’)  R (5’-GGACTACHVGGGTWTCTAAT-3’) |
