# Supplementary material for: Effect of dapagliflozin on ferroptosis through the gut microbiota metabolite TMAO during myocardial ischemia–reperfusion injury in diabetes mellitus rats
Source: Sci Rep. 2024 Jun 15;14:13851. doi: 10.1038/s41598-024-64909-5 (PMC11180094; doi:10.1038/s41598-024-64909-5)
Supplement: Supplementary file 2 — Supplementary Table S2. [file 41598_2024_64909_MOESM2_ESM.docx]

| **Circos plot (top5)** | | | |
| --- | --- | --- | --- |
|  | **N(%)** | **DIR(%)** | **DAPA(%)** |
| **Class** |  |  |  |
| Bacteroidia | 36.80 | 37.51 | 42.21 |
| Clostridia | 42.72 | 23.27 | 19.90 |
| Gammaproteobacteria | 0.31 | 15.62 | 22.42 |
| Bacilli | 4.76 | 13.33 | 7.40 |
| Deltaproteobacteria | 5.77 | 0.97 | 0.93 |
| **Family** |  |  |  |
| Muribaculaceae | 24.93 | 18.04 | 17.74 |
| Prevotellaceae | 8.39 | 17.09 | 21.85 |
| Ruminococcaceae | 18.01 | 12.96 | 10.20 |
| Enterobacteriaceae | 0.04 | 13.68 | 21.11 |
| Lachnospiraceae | 17.67 | 5.11 | 5.70 |
| **Genus** |  |  |  |
| Muribaculaceae_unclassified | 23.67 | 17.40 | 16.66 |
| Escherichia-Shigella | 0.04 | 12.87 | 19.74 |
| Prevotella_9 | 5.77 | 10.61 | 16.16 |
| Lactobacillus | 4.69 | 10.98 | 4.23 |
| Ruminococcaceae_UCG-005 | 4.07 | 3.40 | 2.60 |
| **Order** |  |  |  |
| Bacteroidales | 36.80 | 37.21 | 42.11 |
| Clostridiales | 42.72 | 23.27 | 19.90 |
| Enterobacteriales | 0.04 | 13.37 | 20.94 |
| Lactobacillales | 4.76 | 13.33 | 7.39 |
| Desulfovibrionales | 5.77 | 0.97 | 0.93 |
| **Phylum** |  |  |  |
| Bacteroidetes | 37.09 | 41.06 | 45.06 |
| Firmicutes | 51.22 | 39.30 | 30.31 |
| Proteobacteria | 6.12 | 16.85 | 23.46 |
| Epsilonbacteraeota | 4.19 | 0.28 | 0.14 |
| Actinobacteria | 0.53 | 2.30 | 0.73 |
| **Species** |  |  |  |
| Muribaculaceae_unclassified | 23.67 | 17.40 | 16.66 |
| Escherichia-Shigella_unclassified | 0.04 | 12.87 | 19.74 |
| Prevotella_9_unclassified | 5.77 | 10.61 | 16.16 |
| Ruminococcaceae_UCG-005_unclassified | 4.07 | 3.40 | 2.60 |
| Prevotellaceae_NK3B31_group_unclassified | 0.17 | 4.73 | 2.97 |
