# Supplementary material for: Effect of dapagliflozin on ferroptosis through the gut microbiota metabolite TMAO during myocardial ischemia–reperfusion injury in diabetes mellitus rats
Source: Sci Rep. 2024 Jun 15;14:13851. doi: 10.1038/s41598-024-64909-5 (PMC11180094; doi:10.1038/s41598-024-64909-5)
Supplement: Supplementary file 3 — Supplementary Table S3. [file 41598_2024_64909_MOESM3_ESM.docx]

| **Degree quantity** | | | | |
| --- | --- | --- | --- | --- |
| Name | Degree | Eccentricity | EdgeCount | Indegree |
| ALB | 10 | 1 | 10 | 0 |
| MAPK1 | 5 | 2 | 5 | 1 |
| HMOX1 | 8 | 2 | 8 | 2 |
| PPARG | 10 | 1 | 10 | 9 |
| MAPK8 | 7 | 2 | 7 | 3 |
| PARP1 | 6 | 2 | 6 | 4 |
| CBS | 3 | 2 | 3 | 1 |
| SRC | 7 | 2 | 7 | 7 |
| LCN2 | 3 | 2 | 3 | 2 |
| PPARA | 6 | 2 | 6 | 4 |
| DPP4 | 3 | 2 | 3 | 1 |
